# Changes in litter quality induced by nutrient addition alter litter decomposition in an alpine meadow on the Qinghai-Tibet Plateau

**DOI:** 10.1038/srep34290

**Published:** 2016-10-03

**Authors:** Wenyan Zhu, Jinzhou Wang, Zhenhua Zhang, Fei Ren, Litong Chen, Jin-Sheng He

**Affiliations:** 1 Key Laboratory of Adaptation and Evolution of Plateau Biota, Haibei Alpine Meadow Ecosystem Research Station, Northwest Institute of Plateau Biology, Chinese Academy of Sciences, Xining 810008, China; 2 University of Chinese Academy of Sciences, Beijing 100049, China; 3 Department of Ecology, College of Urban and Environmental Sciences, and Key Laboratory for Earth Surface Processes of the Ministry of Education, Peking University, Beijing 100871, China

## Abstract

The effects of nitrogen (N) and phosphorus (P) addition on litter decomposition are poorly understood in Tibetan alpine meadows. Leaf litter was collected from plots within a factorial N × P addition experiment and allowed to decompose over 708 days in an unfertilized plot to determine the effects of N and/or P addition on litter decomposition. Results showed that nutrient addition significantly affected initial P and P-related biochemical properties of litter from all four species. However, the responses of litter N and N-related biochemical properties to nutrient addition were quite species-specific. Litter C decomposition and N release were species-specific. However, N and P addition significantly affected litter P release. Ratios of Hemicellulose + Cellulose to N and P were significantly related to litter C decomposition; C:N ratio was a determinant of litter N release; and C:P and (Hemicellulose + Cellulose):P controlled litter P release. Overall, litter C decomposition was controlled by litter quality of different plant species, and strongly affected by P addition. Increasing N availability is likely to affect litter C decomposition more indirectly by shifting plant species composition than directly by improving litter quality, and may accelerate N and P cycles, but shift the ecosystem to P limitation.

Plant litter decomposition is a key process regulating carbon (C) storage and nutrient (e.g., N, nitrogen, and P, phosphorus) cycles in most terrestrial ecosystems^1^,^2^. Many studies have shown that litter decomposition usually depends on the environmental conditions, litter quality (i.e., its chemical composition) and decomposer activity^2^,^3^,^4^,^5^,^6^. Under specific climatic conditions, litter quality, e.g., N and P concentrations, and ratios of C:N, C:P and lignin:N are generally thought to control litter C decomposition and nutrient release^5^,^7^,^8^,^9^.

However, litter chemical composition and thus litter decomposition can be strongly affected by changes in nutrient availability induced by human activity and climate change. For instance, increasing atmospheric N deposition has been widely observed on regional to global scales due to increased fossil fuel consumption and fertilizer application^10^,^11^,^12^. In addition, soil organic matter (including organic N and P) mineralization is expected to increase due to global warming^13^,^14^. Such increased nutrient availability could directly affect litter decomposition by improving litter quality (e.g., a higher N and P content, and lower C:N and C:P ratios)^15^,^16^, or indirectly by changing plant species composition^17^,^18^,^19^ and thus the litter’s chemical composition.

Whilst it has been suggested that plant species exert major control on litter decomposition at the global scale^20^, the magnitude of direct or indirect effects of nutrient enrichment may be quite different at the site or regional scale. For instance, in an annual herb-based microcosm ecosystem (with eight species), Manning, *et al.*^21^ found that the direct effects of N addition significantly affected litter decomposition, but the indirect effects did not. However, in two grasslands rich in species (19–22 species m^−2^) Aerts, *et al.*^17^ showed that bulk litter decomposition was not increased by long-term (12-year) nutrient additions, but determined by initial plant species composition and thus by litter quality. These studies suggest that original plant species composition significantly affects the response of litter decomposition to nutrient addition, and further studies at the species level are still needed.

The alpine meadows of the Qinghai-Tibet plateau, an ecologically fragile area, are deficient in available N and P^22^. While litter decomposition could be an important source of nutrients for plant growth, the low temperature caused by the high altitude of this region could be a major limitation for litter decomposition. However, during recent decades, the alpine meadows have experienced increasing N deposition^12^,^23^ and a warming climate^24^,^25^, which could significantly increase nutrient availability and thus affect litter decomposition and nutrient cycles. However, data on the extent to which nutrient enrichment affects litter decomposition at species level are still lacking. Therefore, our objectives were to investigate how nutrient (N and/or P) addition affects quality, C decomposition and nutrient release in aboveground plant litter produced by the dominant species in the alpine meadows of the Qinghai-Tibet plateau.

## Results

### Initial quality of leaf litter

The addition of N and/or P had different effects on initial chemical content and the nutrient ratios of leaves, and the response to nutrient addition was specific to each species. We used generalised linear models (GLMs) to analyse the effect of N addition, P addition, and their interaction (Tables 1 and 2). N addition and P addition and the interaction between the two significantly affected C content in *Kobresia humilis*, *Tibetia himalaica* and *Gentiana straminea (P* < 0.05) but did not affect C in *Stipa aliena (P* > 0.05). Across all four species, N- or P addition affected total C in leaf litter. Total N content in *K. humilis*, *S. aliena* and *G. straminea* was increased (*P* < 0.05) by nutrient addition but this was not the case in *T. himalaica (P* > 0.05) (Tables 1 and 2), and N concentration was not affected by nutrient addition in any of the species (*P* > 0.05).

Generally, the initial litter P content in the four species was significantly (*P* < 0.05) higher after P and NP addition treatments than either after CK or N addition (Table 2). The GLM analysis showed that N- or P addition and N, P interaction significantly (*P* < 0.05) increased the initial P content in leaf litter of the four species (Table 1). The initial P content of *K. humilis*, *T. himalaica* and *G. straminea* after the NP treatment was significantly lower than that after the P addition treatment, whereas the P content of *S. aliena* after the NP treatment was significantly higher than that after P addition alone (Table 2). Although the lignin, cellulose, and hemicellulose content was occasionally significantly (*P* < 0.05) altered in *T. himalaica* and *G. straminea*, their content in *K. humilis* and *S. aliena* was not greatly affected by nutrient addition (Table 1). In the four species, nutrient addition considerably increased the initial P content and decreased lignin content in leaf litter (Table 1).

We used the ratios to give information about relative chemical composition. C:N ratios and (Hemicellulose + cellulose):N ratios were not changed by nutrient addition in any of the species (*P* > 0.05), and lignin:N was not affected in *T. himalaica*. Otherwise, nutrient addition affected ratios in all four species: significantly influencing the C:P ratios, N:P ratios, lignin:P ratios and (Hemicellulose + cellulose):P ratios (*P* < 0.05).

Leaf litter chemical composition and the ratios differed more between species than between nutrient addition treatments (Table 2). The maximum mean total C was in *T. himalaica*, and the variation was relatively small between the four species. Mean total N and P content were higher in *T. himalaica* than in the other three non-legume species. Lignin in *T. himalaica* and *G. straminea* were higher but hemicellulose and cellulose were lower than in *K. humilis* and *S. aliena*.

### Litter C loss

**Figure 1:**
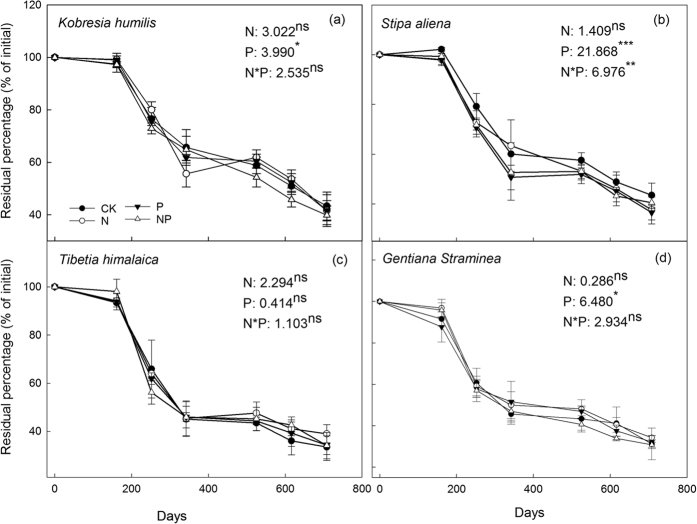
Percentage of C remaining in leaf litter under different nutrient addition treatments in four species: (**a**) Kobresia humilis, (**b**) Stipa aliena, (**c**) Tibetia himalaica, and (**d**) Gentiana straminea. CK: control, N: nitrogen addition, P: phosphorus addition, NP: combined N and P addition. A repeated measures ANOVA was used to analysis the effect of N and P addition, and their interaction on litter C decomposition.

In all four species, the percentage of litter remained stable during the cold season (October to April) and decreased rapidly in the warm season (May to September) (Fig. 1). Overall, the decomposition of litter C was significantly affected by P addition in all the plant species except *T. himalaica*, but this was not the case for N addition. For example, compared with the CK treatment, P addition accelerated the loss of litter C in *K. humilis* and *S. aliena*, but decreased it in *G. straminea*. The interaction of N and P addition only showed significant effect in *S. aliena* (Fig. 1). At the end of the field incubation period, the percentage of C remaining was significantly different between the different plant species, but not between the nutrient addition treatments (Table 3). For instance, the average percentage of C remaining was ranked: *K. humilis* (41.6%) = *S. aliena* (39.5%) > *T. himalaica* (35.2%) = *G. straminea* (32.7%). The decomposition kinetics estimated using the two-pool exponent model showed that the fraction of initial total C was 64–78% in the active C pool with a decomposition rate constant of 0.63–1.80 yr^−1^ and was 22–36% in the stable C pool with no decomposition during the experimental period (Fig. S1, Table 3). Statistics showed that the fraction of initial total C in the active/stable C pool, and the decomposition rate constant for active C were significantly affected by plant species, but not by nutrient addition. The average fraction in the active C pool was greater in *K. humilis* (76.9%) and *S. aliena* (73.0%) than in *G. straminea* (68.4%) and *T. himalaica* (66.5%). However, the decomposition rate constant for the active C pool was lower in *K. humilis* (0.66 yr^−1^) and *S. aliena* (0.87 yr^−1^) than in *G. straminea* (1.51yr^−1^) and *T. himalaica* (1.58 yr^−1^) (Table 3). The estimated fraction of the stable C pool in the initial total C was much lower than the observed percentage of C remaining at the end of the experiment in *K. humilis* and *S. aliena* (23.1–27.0% vs. 39.5–41.6%), but similar to the observed value in *T. himalaica* and *G. straminea* (32.7–35.2% vs. 31.6–33.5%), suggesting that material from the former two plant species will continue to decompose quickly, while the later two had reached a stable level and will decompose more slowly subsequently.

### Litter N and P release

**Figure 2:**
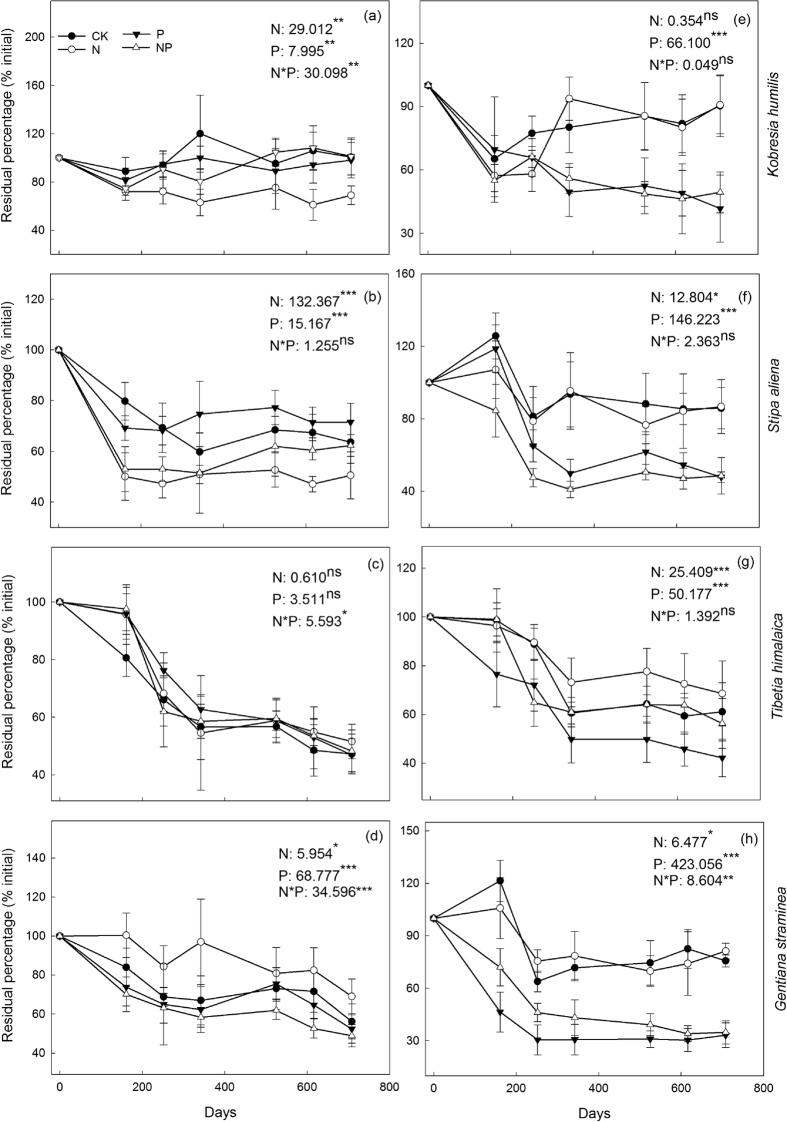
Residual percentages of initial N (**a–d**) and P (**e–h**) in leaf litter under different nutrient addition treatments in the four plant species. A repeated measures ANOVA was used to analysis the effect of N and P addition, and their interaction on residual nutrient. CK: control, N: nitrogen addition, P: phosphorus addition, NP: combined N and P addition.

The pattern of N gain or loss in leaf litter during the experimental period was significantly affected by plant species and nutrient addition (Fig. 2a–d and Fig. S2). The percentage of N remaining was significantly affected by N addition and P addition in *K. humilis*, *S. aliena* and *G. straminea*, but not in *T. himalaica*, and by the interaction of N and P addition in all the plant species except *S. aliena*. In detail, the average percentage of N remaining at the end of the experiment was similar to the value of the stable level estimated by two-pool models, and the species could be ranked: *K. humilis* (92.2%) >* S. aliena* (62.0%) = *G. straminea* (56.6%) > *T. himalaica* (48.5%) (Table 3). In *K. humilis*, the percentage of N remaining slightly decreased in the first few months and increased thereafter to 98–101% at the end of the experiment under the CK, P and NP treatments, but decreased to a stable value of 69% in the N treatment (Table 3). In *S. aliena*, and *G. straminea*, N release exhibited the same pattern, i.e., a sharp decrease during the first few months, and stable thereafter. While net N release was also found in *T. himalaica*, it took more than a year to reach a stable level. Interestingly, the percentage of N remaining at the end of the experiment was significantly lower in the N treatment (50.5%) than in the CK, P and NP treatments (62.3–71.5%) for *S. aliena*, but greater for *G. straminea* (69.1% vs. 48.9–56.2%) and similar for *T. himalaica* (51.6% vs. 46.9–48.3%) (Table 3).

Generally, the percentage of P remaining was significantly affected by N and P addition in all the plant species (Fig. 2e–h and Fig. S3). One exception was that N addition did not have a significant effect on P release in *K. humilis*. However, the significant interactive effect of N and P addition on P release was only found in *G. straminea*, and not in the other three species. The percentage of P remaining at the end of the experiment was greater in the CK and N treatments (61–91%) than in the P and NP treatments (33–56%).

### Relationships between biochemical composition and litter decomposition

Biochemical components were significantly related to the decomposition of leaf C, and nutrient release (Tables 4 and 5). Linear regression showed that the C:N ratio, lignin content, cellulose + hemicellulose, and ratios of cellulose + hemicellulose to N and P were significantly related to the fractions of active and stable C pools in the initial total C pool and the decomposition rate constant of the active C pool (Table 4). The ratios of total C, lignin, and cellulose + hemicellulose to N were significantly related to the percentage of N in the stable level. Similarly, those organic components to P ratios were significantly related to the percentage of P in the stable level (Table 4). Nevertheless, multi-step regression analysis showed that the decomposition rate constant of the active C pool was mainly controlled by the ratios of cellulose + hemicellulose to nutrient (i.e., N and P) (Table 5). The fraction in the stable pool was controlled by (Hemicellulose + Cellulose):N ratio with respect to the C remaining, by C:N ratio with respect to the N remaining, and by C:P and (Hemicellulose + Cellulose):P ratios with respect to the P remaining (Table 5).

## Discussion

### Litter quality and litter C decomposition

In line with previous findings^20^, plant species significantly affected the decomposition of litter C (Fig. 1 and Table 3); this could be attributed to differences in litter quality. In general, initial lignin content and lignin:N ratio are considered to be the major factors controlling the decomposability of litter, especially in woody plant species^7^,^20^,^26^. In a path analysis, Zhang, *et al.*^3^ found that the C:N ratio and total nutrient content (the sum of individual nutrient concentrations) of the litter were the two factors that had the greatest effect on litter mass loss across a wide range of plant species and tissues. However, in the alpine meadow studied here, we found that (Hemicellulose + Cellulose):N ratio was the mostly direct factor on the participation of litter C components and the decomposition rate constant of active C (Table 5).

Despite its strong influence on initial N content and the ratios of C, lignin, and hemicellulose/cellulose to N (especially in *K. humilis* and *S. aliena*), N addition did not significantly affect the decomposition of litter C associated with any of the four plant species (Table 1 and Fig. 1). This result suggests a neutral direct effect of increasing atmospheric N deposition on litter C decomposition in this region. However, in a meta-analysis of the effects of N addition on litter decomposition, Knorr, *et al.*^27^ found that decomposition was inhibited by N addition for low-quality (lignin content > 20%) litters, but accelerated for high-quality (lignin content < 10%) litters. Nevertheless, we did not find a significantly positive effect of N addition on litter C decomposition, even though all four plant species in our study (4–8% lignin content) produced high-quality litters (Table 2). On the one hand, the increased N supply could increase the proportion of litter humification rather than decomposition, e.g., by suppressing the activity of lignolytic enzymes^28^,^29^, or increasing the microbial C use efficiency^30^,^31^. On the other hand, the quality of leaf litter in our study was within the ideal (non-limiting) levels (i.e., lignin content <10% and lignin:N < 10) for decomposition as summarised by Prescott^4^, suggesting that other nutrient content (e.g., P), or climatic conditions (e.g., temperature) could be major limiting factors for litter decomposition. For example, the mean annual temperature at this site (−1.6 °C) was less than the threshold value (10 °C), and could inhibit the overall decay process^4^.

Unlike N addition, P addition significantly accelerated the decomposition of non-legume litter (i.e., litter of *K. humilis*, *S. aliena*, *G. straminea*) but not the legume litter (i.e. of *T. himalaica*) (Fig. 1). This was probably due to the improving stoichiometry of C and nutrients. For example, the C:P ratios of non-legume litter decreased from 773–810 in the control and N addition treatments to 241–359 in the P and NP treatments (Table 2). A wealth of evidence shows that there is a strong stoichiometry of C:N:P in soil humus (e.g., 1000:83.3:20, Himes^32^) and microbial biomass (e.g., 1000:116.7:16.7; Creamer *et al.*, 2014), and the stoichiometry controls litter C decomposition^9^. The initial C:N:P ratio of the three non-legume litters was 1000:(17.0–29.1):(1.2–1.3) under the no-fertilizer control. If one third of the initial C was finally converted to stable humus-C, the initial nutrient content could only support a ratio of 1000:(50.9–87.2):(3.7–3.9) in humus when N supply was very close to the demand of humus formation; however, the P supply was only able to fulfil a quarter of the demand. These results suggest that P was more limited than N with respect to litter C decomposition for the three non-legume plant species. At the end of the experiment, the percentage of C remaining was not affected by P addition (Table 3), suggesting a counterbalance between the positive effect of P addition to litter C decomposition and humification. Nevertheless, for *S. aliena* (a dominant species accounting for 31.5% of the community aboveground biomass), P addition tended to accelerate the decomposition of active C, but to increase the proportion of stable C (Table 3). In addition, the N:P ratios were generally greater in leaf litter under the no P addition treatment (20.5–28.8) than the critical value of 16 reported by Koerselman and Arthur^33^, suggesting that P availability limited plant growth in this region. Therefore, long-term P addition could promote litter C sequestration in this region both by increasing plant biomass and thus C input, and by increasing litter C humification.

### Nutrient release

Unlike the patterns of litter C loss, net N release and immobilization in leaf litter were strongly controlled by initial C:N ratio (Fig. 2a–d and Table 5). For instance, the N-fixing legume *T. himalaica*, with a low C:N ratio (15.2–16.4, Table 2), exhibited continuous N release during the experimental period, because the N requirements of microbial decomposers were being met. The non-legume plants *S. aliena* and *G. straminea*, with a median C:N ratio (26.8–39.5, Table 2), exhibited rapid N release during the first few months but no net N immobilisation or release thereafter. However, the non-legume plant *K. humilis* with a high C:N ratio (47.2–58.9, Table 2) showed little or no net N immobilisation/release. Our results are in line with those of Parton, *et al.*^8^ that net N release occurred when the average C:N ratio of the leaf litter was <40 (a range of 31 to 48). As the C:N ratios of major plant species were generally less than 40 in the alpine meadows of the Qinghai-Tibet plateau (Table 2, and supplemental Table 1 in Duan, *et al.*^34^), the future increasing N deposition in this region may not change the pattern but may change the magnitude of initial N release.

The pattern of P release was strongly controlled by C:P and (Hemicellulose + Cellulose):P ratios (Table 5), and significantly regulated by P addition (Fig. 2). When the initial C:P ratios were within the critical values for P release (700–900) observed by Moore, *et al.*^35^, e.g., litter of non-legume plant without P addition, we only found limited P immobilisation at the beginning of the experiment and little P loss thereafter. However, when the initial C:P ratio was lower than the critical values, e.g., litter of a legume plant with/without P addition and non-legume plant with P addition, P was immediately lost from the litter of all plant species and the magnitude of P loss was much greater.

## Implications

Our study showed that the variation in litter C decomposition was much greater between plant species than between different nutrient additions (Table 3), suggesting that any management without a shift in plant species composition (and thus litter quality) is unlikely to affect the litter C decomposition significantly. Many studies have shown that increasing nutrient (especially N) addition could increase plant biomass, but decrease plant species diversity and cause shifts in plant community composition^15^,^16^,^36^,^37^. In the same ecosystem near to our experimental site, Yang, *et al.*^38^ found that N and P additions significantly increased the dominance of graminoids (including *S. aliena*), but decreased the dominance of forbs (including *T. himalaica* and *G. straminea*) in the community after 4 years of fertilizer application. Taken together, our results and those demonstrating plant species changes resulting from nutrient addition^38^ suggested that the indirect effect of nutrient addition (i.e., changing the plant community composition) was more important than the direct effect (i.e., changing the litter quality) on litter C decomposition on the Qinghai-Tibet plateau. While future increasing N deposition in this region may increase the overall plant biomass and thus mean more highly N-enriched litter C input, the effect of P limitation on plant growth and microbial C use efficiency will restrict C sequestration in litter. Therefore, to maximise sequestering C and N in the soil-plant system of alpine meadows, a certain amount of P should be added.

## Material and Methods

### Experimental site

This study was conducted at the Haibei Alpine Meadow Ecosystem Research Station (latitude: 37° 37′ N, longitude: 101° 12′ E; elevation: 3200 m above sea level) in Qinghai Province; this area is characterised by a continental monsoon climate with severe, long winters and short, cool summers. The mean annual temperature and precipitation are −1.6 °C and 500 mm, respectively; 80% of precipitation falls in the growing seasons from May to September. The soil is classified as Mat-GryicCambisol (according to Chinese Soil Taxonomy), with pH 7.46, total organic C 72.4 g kg^−1^, total N 8.7 g kg^−1^, total P 0.84 g kg^−1^ and available P 6.2 mg kg^−1^ in the 0–5 cm soil layer (Table 6). The plant species in the alpine meadows are divided into four functional groups: (I) sedges: *K. humilis*, *Carex scabrirostris,* etc; (II) graminoids: *S. aliena*, *Elymus nutans*; *Poa pratensis*, etc; (III) leguminous forbs: *T. himalaica*, *Medicago archiducis-nicolai,* etc; and (IV) non-leguminous forbs: *G. straminea*, *Potentilla nivea*, *Saussurea superba*, etc. The corresponding dominant species in each functional group were *K. humilis*, *S. aliena*, *T. himalaica* and *G. straminea*, accounting for 3.1%, 31.5%, 5.0% and 5.0% of the community aboveground biomass, respectively.

### Fertilisation design

A 110 m × 75 m area of flat land with relatively uniform vegetation was fenced in May 2009. A two-way factorial design with N and P addition was implemented involving four treatments: (1) CK, control; (2) N, nitrogen addition (10 g Nm^−2^yr^−1^ as urea); (3) P, phosphorus addition (10 g Pm^−2^yr^−1^ as triple superphosphate); and (4) NP, combined N and P addition (10 g N m^−2^yr^−1^ + 10 g P m^−2^yr^−1^). All nutrients were applied twice per year (50% on June 22^nd^ and 50% on July 15^th^) after sunset, when the soil moisture was high, in both 2009 and 2010. In total, 20 plots (measuring 6 m × 6 m with a 2-m wide buffer zone), comprising 4 treatments with 5 replicates each, were used in a completely randomised design at the study site.

### Litter-bag incubation experiment

In early October of 2010, freshly senesced leaf litters were sampled from the four dominant species (*K. humilis*, *S. aliena*, *T. himalaica*, and *G. straminea*) grown under each of the different treatments. The litters were air-dried until they reached a constant weight, then placed in polyethylene litter bags (10 cm × 15 cm, with an upper layer of 1 mm × 1 mm mesh and a lower layer of 0.5 mm × 0.5 mm mesh). Each litter bag was filled with 4 g of air-dried litter, clipped to fragments measuring 5 cm in length. The litter bags were placed in a plot that had not received any fertilizer treatment, and the bags were staked on the soil among the senescent forbs for 708 days from 10 November 2010 to 20 October 2012. The litter bags were collected twice during the growing season (April 20 and July 20 in 2011 and 2012) and once outside the growing season (October 20 of 2011 and 2012) in each year. In total, 480 litter bags (4 treatments applied to 4 species across 5 replicates with 6 collection and measurement points) were prepared and deployed onto the treatment plots. Another 80 litter bags (4 treatments applied to 4 species across 5 replicates) were prepared using the same initial drying treatment and directly brought to the laboratory for the measurement of weight after oven drying and determination of initial chemistry.

### Litter chemical analysis

The initial chemical composition of all plant litter was determined for subsamples. The retrieved litter samples were oven-dried at 65 °C for 48 h and weighed. Next, carbon (C) and N content of each leaf litter sample was assayed using an elemental analyser (2400 II CHNS/O Elemental Analyzer; Perkin-Elmer, Boston, MA, USA). Total P was determined using the molybdate colorimetric method after ascorbic acid reduction^39^. Lignin, cellulose, and hemicellulose were determined using the sulphuric acid procedure^40^. The C:N ratio and initial P, lignin, cellulose, and hemicellulose content were used to represent the leaf litter quality.

### Data analysis

Litter decomposition rate varied across the seasons, e.g., faster in the warm season (April to September) and slower or even zero in the cool season (October to March). To reduce the bias in simulation of litter decomposition, we used a standard year which was modified on the basis of cumulative degree days (CDD). CDD was calculated by summing >0 °C degree days from the date litter was placed in the field to the date of sampling. Daily temperature <0 °C was set to 0. A standard year was defined to be 1723 cumulative degree days at the experimental site, which was the average value of a period lasting 30 years (1981–2010). Therefore, the seven sampling dates were on the standard year of 0, 0.05, 0.58, 1.06, 1.09, 1.64 and 2.14, respectively. The percentage of litter C, N and P remaining was estimated by fitting a two-pool model (Fig. S1–S3):


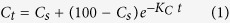



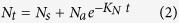



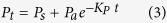


where *C*_*t*_, *N*_*t*_ and *P*_*t*_ are the percentage of litter C, N and P remaining at time *t* (standard years), respectively. *C*_*s*_, *N*_*s*_ and *P*_*s*_ are the fractions in the stable pools, and 100 − *C*_*s*_, *N*_*a*_ and *P*_*a*_ are the fractions in the active pools for litter C, N and P, respectively. *K*_*C*_, *K*_*N*_ and *K*_*P*_ are the decomposition rate constants of litter C, N and P in the active pools.

GLMs with multi-comparisons were used to determine the effects of plant species and nutrient addition (N and P addition and their interactions) on all the considered initial litter quality, and percentage of litter C, N and P remaining at the end of the experiment. Repeated measures ANOVA was used to analyse the effect of N, P and N, P interaction on litter C decomposition and nutrient release; the analyses were performed with R 3.1.1. Linear regression was used to test and verify the correlation between the litter quality parameters (total C, lignin, cellulose and hemicellulose, and their ratios to N and P) and the litter C decomposition or nutrient release. Statistical analyses were performed using SPSS 16.0.

## Additional Information

**How to cite this article**: Zhu, W. *et al.* Changes in litter quality induced by nutrient addition alter litter decomposition in an alpine meadow on the Qinghai-Tibet Plateau. *Sci. Rep.* **6**, 34290; doi: 10.1038/srep34290 (2016).

## Supplementary Material

Supplementary Information

## Figures and Tables

**Table 1:** Summary of GLMs (*F*-value) for the effects of nitrogen addition (N), phosphorus addition (P), and N and P interaction (N × P) on the chemical properties of leaf litter.

		Total C	Total N	Phosphorus	Lignin	Cellulose	Hemicellulose
All species							
N		4.79*	0.87^ns^	7.26^**^	2.19^ns^	0.05^ns^	0.09^ns^
P		6.92*	0.00^ns^	168.89^***^	4.94*	0.01^ns^	0.10^ns^
N × P		0.09^ns^	0.41^ns^	4.08*	0.02^ns^	0.12^ns^	0.06^ns^
*K. humilis*							
N		0.04^ns^	17.22^***^	20.22^***^	0.94^ns^	2.04^ns^	3.78^ns^
P		1.53^ns^	9.01^**^	238.38^***^	1.17^ns^	3.55^ns^	1.85^ns^
N × P		27.32^***^	48.54^***^	16.00^***^	2.17^ns^	0.82^ns^	2.08^ns^
*S. aliena*							
N		0.21^ns^	64.05^***^	16.76^***^	0.23^ns^	0.001^ns^	0.81^ns^
P		0.02^ns^	7.77*	580.1^***^	0.01^ns^	0.19^ns^	0.74^ns^
N × P		1.37^ns^	7.12*	11.58^**^	0.33^ns^	1.27^ns^	0.09^ns^
*T. himalaica*							
N		11.54^**^	0.08^ns^	17.34^***^	0.00^ns^	0.009^ns^	0.72^ns^
P		16.90^***^	2.37^ns^	85.96^***^	2.18^ns^	7.11*	1.11^ns^
N × P		25.03^***^	1.45^ns^	5.67*	6.95*	2.47^ns^	6.30*
*G. straminea*							
N		7.00*	1.14^ns^	7.48*	13.90^**^	1.54^ns^	0.96^ns^
P		5.18*	10.29^***^	123.75^***^	20.50^***^	0.57^ns^	4.94*
N × P		0.53^ns^	0.85^ns^	6.42*	1.84^ns^	3.99^ns^	0.02^ns^
	C: N	C:P	N:P	Lignin:N	Lignin:P	(H+C):N	(H+C):P
All species							
N	2.57^ns^	0.903^ns^	19.750^***^	4.386*	0.057^ns^	1.362^ns^	0.152^ns^
P	0.01^ns^	183.129^***^	230.160^***^	1.201^ns^	121.731^***^	0.052^ns^	49.274^***^
N × P	1.91^ns^	0.260^ns^	3.607^ns^	2.430^ns^	1.233^ns^	0.910^ns^	0.013^ns^
*K. humilis*							
N	9.54^**^	3.023^ns^	12.283^**^	7.966*	0.402^ns^	7.424*	3.560^ns^
P	2.37^ns^	62.347^***^	36.673^***^	0.002^ns^	46.155^***^	0.989^ns^	63.446^***^
N × P	45.20^***^	2.825^ns^	1.957^ns^	26.582^***^	4.283^ns^	15.653^***^	1.891^ns^
*S. aliena*							
N	80.67^***^	0.030^ns^	5.865*	26.670^***^	0.019^ns^	59.760^***^	0.054^ns^
P	6.03*	49.071^***^	37.005^***^	2.786^ns^	35.454^***^	3.007^ns^	49.550^***^
N × P	3.87^ns^	0.440^ns^	0.706^ns^	0.689^ns^	0.145^ns^	2.902^ns^	0.592^ns^
*T. himalaica*							
N	1.07^ns^	11.093^**^	11.360^**^	0.053^ns^	10.217^**^	0.116^ns^	10.699^**^
P	6.26*	55.868^***^	38.603^***^	3.396^ns^	41.808^***^	6.472*	48.703^***^
N × P	0.11^ns^	0.103^ns^	0.115^ns^	2.649^ns^	0.327^ns^	1.336^ns^	0.028^ns^
*G. straminea*							
N	2.09^ns^	2.229^ns^	5.009*	15.776^***^	0.493^ns^	0.101^ns^	2.035^ns^
P	13.69^**^	65.948^***^	78.167^***^	33.101^***^	71.664^***^	4.618*	43.447^***^
N × P	0.88^ns^	1.824^ns^	2.267^ns^	1.006^ns^	6.774*	0.223^ns^	0.542^ns^

^*^P ≤ 0.05, ^**^P ≤ 0.01, ^***^P ≤ 0.001, ns: not significant, (H + C): Hemicellulose + cellulose.

**Table 2:** Mean biochemical composition of leaf litter after different nutrient addition in the four species.

Species	Treatment	Total C (%)	Total N (%)	Phosphorus (‰)	Lignin (%)	Cellulose (%)	Hemicellulose (%)	
*K. humilis*	CK	45.2 ± 0.41a	0.78 ± 0.09b	0.59 ± 0.08c	6.00 ± 1.09a	29.0 ± 2.55b	34.6 ± 1.13a	
	N	44.0 ± 0.34b	1.20 ± 0.09a	0.56 ± 0.04c	5.21 ± 0.52a	30.6 ± 1.00ab	32.9 ± 1.15b	
	P	43.6 ± 0.90b	0.93 ± 0.07b	1.77 ± 0.25a	5.17 ± 0.41a	30.9 ± 0.95ab	33.2 ± 1.26ab	
	NP	44.9 ± 0.38a	0.82 ± 0.09b	1.25 ± 0.07b	5.34 ± 0.70a	31.3 ± 0.76a	33.0 ± 0.74b	
	**Mean**	**44.4** ± **0.85AB**	**0.93** ± **0.19C**	**1.05** ± **0.53B**	**5.4** ± **0.75B**	**30.4** ± **1.63B**	**33.4** ± **1.21A**	
*S. aliena*	CK	44.1 ± 0.35a	1.13 ± 0.07c	0.55 ± 0.07c	3.91 ± 0.51a	33.0 ± 0.77a	34.6 ± 0.82a	
	N	44.3 ± 0.98a	1.67 ± 0.15a	0.58 ± 0.05c	4.15 ± 0.50a	32.6 ± 0.62a	34.4 ± 0.64a	
	P	44.4 ± 0.76a	1.13 ± 0.09c	1.33 ± 0.09b	4.06 ± 0.53a	32.7 ± 0.83a	34.4 ± 0.77a	
	NP	43.9 ± 0.28a	1.39 ± 0.12b	1.61 ± 0.12a	4.04 ± 0.54a	33.1 ± 0.84a	34.0 ± 1.19a	
	**Mean**	**44.2** ± **0.64B**	**1.33** ± **0.25B**	**1.02** ± **0.48B**	**4.0** ± **0.49C**	**32.8** ± **0.74A**	**34.4** ± **0.85A**	
*T. himalaica*	CK	44.9 ± 0.30a	2.74 ± 0.15a	1.25 ± 0.18c	6.01 ± 0.36ab	20.1 ± 0.96ab	17.4 ± 1.20b	
	N	45.2 ± 0.41a	2.84 ± 0.18a	1.08 ± 0.12c	6.71 ± 0.51a	21.1 ± 1.12a	18.2 ± 0.91ab	
	P	45.1 ± 0.50a	2.93 ± 0.20a	2.40 ± 0.38a	6.32 ± 0.90ab	19.4 ± 2.00ab	19.0 ± 1.23a	
	NP	43.5 ± 0.46b	2.87 ± 0.08a	1.76 ± 0.05b	5.61 ± 0.49b	18.3 ± 1.68b	17.5 ± 0.65b	
	**Mean**	**44.7** ± **0.81A**	**2.85** ± **0.16A**	**1.62** ± **0.57A**	**6.2** ± **0.69A**	**19.7** ± **1.74C**	**18.0** ± **1.15B**	
*G. straminea*	CK	45.4 ± 0.69a	1.33 ± 0.09b	0.58 ± 0.03c	8.32 ± 0.86a	12.5 ± 0.98b	14.5 ± 2.49a	
	N	44.9 ± 0.93a	1.33 ± 0.11b	0.56 ± 0.04c	6.31 ± 0.65b	14.5 ± 1.55a	13.7 ± 1.93ab	
	P	45.0 ± 0.31a	1.45 ± 0.13ab	1.93 ± 0.40a	6.00 ± 1.11bc	13.3 ± 1.58ab	12.5 ± 1.33ab	
	NP	44.0 ± 0.47b	1.56 ± 0.15a	1.41 ± 0.17b	5.06 ± 0.85c	12.8 ± 1.37ab	11.4 ± 2.70b	
	**Mean**	**44.8** ± **0.79A**	**1.42** ± **0.15B**	**1.12** ± **0.63B**	**6.4** ± **1.46A**	**13.3** ± **1.50D**	**13.0** ± **2.34C**	
Species	Treatment	C: N Ratio	C:P	N:P	Lignin:N	Lignin:P	(H+C):N	(H+C):P
*K. humilis*	CK	58.9 ± 6.17a	772.6 ± 88.61a	13.3 ± 2.46b	7.8 ± 1.25a	102.8 ± 22.77a	83.0 ± 11.91a	1090.1 ± 154.40a
	N	36.7 ± 2.40c	785.8 ± 60.13a	21.5 ± 2.48a	4.4 ± 0.63c	92.7 ± 7.55a	53.0 ± 3.58c	1134.5 ± 67.48a
	P	47.2 ± 3.02b	250.7 ± 38.00c	5.4 ± 1.07c	5.6 ± 0.37bc	29.9 ± 6.79b	69.5 ± 6.72b	368.1 ± 48.90c
	NP	55.4 ± 6.50a	359.2 ± 20.48b	6.5 ± 0.75c	6.6 ± 1.25ab	42.6 ± 5.97b	79.2 ± 9.45ab	513.5 ± 31.61b
	**Mean**	**49.5** ± **9.91A**	**542.1** ± **252.11A**	**11.68** ± **6.82B**	**6.1** ± **1.56A**	**67.0** ± **34.16AB**	**71.2** ± **14.21A**	**776.6** ± **358.31A**
*S. aliena*	CK	39.0 ± 2.18a	811.0 ± 91.98a	20.9 ± 3.13b	3.5 ± 0.51a	72.0 ± 13.28a	59.8 ± 3.48a	1242.3 ± 132.3a
	N	26.8 ± 2.25c	772.8 ± 54.21a	28.9 ± 1.48a	2.5 ± 0.24b	72.1 ± 6.16a	40.5 ± 3.55c	1169.3 ± 101.19b
	P	39.5 ± 2.71a	335.7 ± 25.10b	8.5 ± 0.96b	3.6 ± 0.32a	30.7 ± 4.65b	59.8 ± 4.65a	507.4 ± 31.41c
	NP	31.7 ± 2.80b	274.0 ± 21.28b	8.7 ± 0.83b	2.0 ± 0.31b	25.1 ± 2.92b	48.5 ± 5.36b	418.5 ± 37.42c
	**Mean**	**34.2** ± **5.92B**	**548.4** ± **256.40A**	**16.8** ± **9.03**A	**3.11** ± **0.56C**	**50.0** ± **23.84BC**	**52.1** ± **9.29B**	**834.4** ± **391.53A**
*T. himalaica*	CK	16.4 ± 0.78a	364.8 ± 51.48b	22.2 ± 3.16b	2.2 ± 0.17ab	49.1 ± 9.79b	13.7 ± 0.44a	304.5 ± 42.07b
	N	15.9 ± 0.92ab	424.6 ± 45.44a	26.6 ± 2.57a	2.4 ± 0.09a	62.8 ± 5.66a	13.8 ± 0.70a	369.5 ± 49.42a
	P	15.4 ± 1.00ab	191.1 ± 26.29d	12.5 ± 2.33d	2.2 ± 0.43ab	26.4 ± 1.87c	13.2 ± 1.34ab	162.3 ± 18.21c
	NP	15.2 ± 0.36b	247.9 ± 8.25c	16.3 ± 0.84c	2.0 ± 0.22b	31.9 ± 2.67c	12.5 ± 0.85b	204.0 ± 14.60c
	**Mean**	**15.7** ± **0.89C**	**307.1** ± **100.63B**	**19.42** ± **5.97A**	**2.2** ± **0.28D**	**42.5** ± **15.69C**	**13.3** ± **0.99D**	**260.1** ± **89.53B**
*G. straminea*	CK	34.4 ± 2.26a	784.7 ± 34.85a	22.9 ± 1.72a	6.3 ± 0.76a	144.1 ± 20.08a	20.6 ± 3.82a	468.3 ± 75.72a
	N	33.8 ± 2.49a	803.7 ± 43.32a	23.8 ± 1.04a	4.7 ± 0.42b	112.7 ± 7.63b	21.2 ± 2.69a	506.4 ± 74.11a
	P	31.2 ± 2.58ab	241.1 ± 46.39b	7.8 ± 1.80c	4.2 ± 1.00bc	31.4 ± 4.08c	18.0 ± 3.27ab	137.0 ± 25.37b
	NP	28.5 ± 2.92b	315.7 ± 32.68b	11.3 ± 2.10b	3.2 ± 0.45c	36.6 ± 9.14c	15.5 ± 1.93b	175.0 ± 40.31b
	**Mean**	**32.0** ± **3.38B**	**536.3** ± **268.58A**	**16.44** ± **7.38A**	**4.6** ± **1.31B**	**81.2** ± **50.95A**	**18.8** ± **3.61C**	**321.7** ± **179.20B**

Different lowercase letters in a column of the same plant species indicate significant difference at *P* ≤ 0.05 in treatment; different capital letters in the same column indicate significant difference in species. CK: Control, N: nitrogen addition, P: phosphorus addition, NP: combined N and P addition.

**Table 3:** Percentage of initial C, N and P remaining in leaf litter after 708 days, and values at stable level estimated by a two-pool model.

Plant species	Treatment	Observed after 708 days			Estimated by a two-pool model			
		f(C)	f(N)	f(P)	*K*_*C*_	*C*_*S*_	*N*_*S*_	*P*_*S*_
*K. humilis*	CK	43.3a	100.6a	90.3a	0.64	23.9	105.3	90.4
	N	41.6a	68.9b	90.8a	0.63	22.6	68.1	96.3
	P	41.6a	98.1a	41.7b	0.65	23.7	95.4	41.1
	NP	39.8a	101.2a	49.4b	0.70	22.3	100.0	47.0
	**Mean**	**41.6 A**	**92.2 A**	**68.1 A**	**0.66 A**	**23.1 A**	**92.2 C**	**68.7 A**
*S. aliena*	CK	43.5a	63.6a	85.8a	0.68	24.4	65.7	86.2
	N	37.6a	50.5b	86.8a	0.78	23.8	49.7	84.3
	P	36.5a	71.5a	47.8b	1.00	28.8	72.1	48.6
	NP	40.4a	62.3a	48.6b	1.02	31.1	57.0	46.8
	**Mean**	**39.5A**	**62.0 B**	**67.2 A**	**0.87 A**	**27.0 AB**	**61.1 AB**	**66.5 A**
*T. himalaica*	CK	33.5a	47.3a	61.1ab	1.48	30.3	48.3	51.9
	N	39.0a	51.6a	68.6a	1.57	35.9	51.3	60.7
	P	34.3a	46.9a	42.2b	1.47	31.9	37.7	35.9
	NP	34.1a	48.3a	56.3ab	1.80	35.8	50.9	60.1
	**Mean**	**35.2 B**	**48.5 C**	**57.0 A**	**1.58 B**	**33.5 B**	**47.1 A**	**52.1 A**
*G. straminea*	CK	33.9a	56.2b	75.7a	1.62	33.6	67.3	73.7
	N	34.2a	69.1a	81.2a	1.43	33.0	67.8	75.3
	P	32.1a	52.3b	33.2b	1.50	31.8	63.9	31.1
	NP	30.8a	48.9b	34.8b	1.48	28.0	56.9	39.4
	**Mean**	**32.7 C**	**56.6 B**	**56.2 A**	**1.51 B**	**31.6 B**	**64.0 B**	**54.9 A**

f(C), f(N) and f(P) were the percentage of initial litter C, N and P remaining at the end of the experiment (%). *K*_*C*_ was the decomposition rate constant of the active C pool (yr^−1^). *C*_*S*_, *N*_*S*_ and *P*_*S*_ were the fraction of litter C, N and P in the stable pools, respectively (%).

**Table 4:** Correlation coefficients (R^2^) between biochemical composition and C decomposition, residual N and P at stable level across the four species.

Biochemical composition	*K*_*C*_	*Cs*	*Ns*	*Ps*
C:N ratio	0.57^***^	0.50^**^	0.90^***^	0.06
C:P ratio	0.12	0.10	0.04	0.86^***^
Lignin	0.34^*^	0.27^*^	0.00	0.00
Lignin:N ratio	0.21	0.20	0.82^***^	0.07
Lignin:P ratio	0.00	0.00	0.04	0.59^***^
Hemicellulose + Cellulose	0.80^***^	0.52^**^	0.19	0.11
(Hemicellulose + Cellulose):N	0.85^***^	0.65^***^	0.68^***^	0.08
(Hemicellulose + Cellulose):P	0.55^***^	0.44^**^	0.09	0.76^***^

*K*_*C*_ was the decomposition constant of active litter C pools; *C*_*S*_, *N*_*S*_ and *P*_*S*_ were the fraction of litter C, N and P in the stable pool.

**Table 5:** Multi-step regression analysis of C decomposition, residual N and P at stable level with biochemical compositions across the four species.

Model	F	P	Adjusted R^2^	df
*K*_*C*_ = −0.012 × (Hemicellulose + Cellulose):N − 0.34 × (Hemicellulose + Cellulose):P × 10^−3^ + 1.82	63.88	0.000	0.89	15
*Cs* = −0.15 × (Hemicellulose + Cellulose):N + 34.6	26.07	0.000	0.63	15
*Ns* = 1.36 × C:N + 21.3	130.70	0.000	0.90	15
*Ps* = 0.054 × C:P + 0.019 × (Hemicellulose + Cellulose):P + 24.1	57.22	0.000	0.88	15

*K*_*C*_ is the decomposition rate constant of the active C pool. *Cs*, *Ns*, *Ps* are the percentage of initial C, N and P in the stable pool, respectively.

**Table 6:** Summary of soil characteristics at different depths at the study site.

Depth	pH	TC (g·kg^−1^)	TOC (g·kg^−1^)	TN (g·kg^−1^)	TP (g·kg^−1^)	AP (mg·kg^−1^)
0–5 cm	7.46 (0.04)	89.0 (1.33)	72.4 (0.16)	8.7 (0.12)	0.84 (0.006)	6.2 (0.009)
5–10 cm	7.61 (0.03)	66.9 (0.72)	62.4 (0.25)	6.9 (0.08)	0.69 (0.006)	5.2 (0.008)
10–20 cm	7.96 (0.02)	54.8 (0.46)	42.8 (0.16)	5.9 (0.07)	0.56 (0.018)	3.2 (0.008)
20–40 cm	8.36 (0.01)	40.4 (0.28)	33.0 (0.19)	3.7 (0.07)	0.44 (0.006)	2.2 (0.011)
40–60 cm	8.57 (0.02)	30.6 (0.29)	23.6 (0.11)	1.9 (0.06)	0.30 (0.011)	0.2 (0.006)

Means of pH, total carbon content (TC), total organic carbon (TOC), total nitrogen concentration (TN), total phosphorous content (TP), and available phosphorous (AP) are shown within parentheses.
