# Supplementary material for: Changes in litter quality induced by nutrient addition alter litter decomposition in an alpine meadow on the Qinghai-Tibet Plateau
Source: Sci Rep. 2016 Oct 3;6:34290. doi: 10.1038/srep34290 (PMC5046129; doi:10.1038/srep34290)
Supplement: Supplementary Information [file srep34290-s1.pdf]

Changes in litter quality induced by nutrient addition alter litter decomposition in an alpine meadow on the Qinghai-Tibet Plateau

Wenyan Zhu, Jinzhou Wang, Zhenhua Zhang, Fei Ren, Litong Chen, JinSheng He

Fig. S1 Percentage of C remaining in leaf litter under different nutrient addition treatments in four species: (a-d) *Kobresia humilis*, (e-h) *Stipa aliena*, (i-l) *Tibetia himalaica*, and (m-p) *Gentiana straminea.* CK: control, N: nitrogen addition, P: phosphorus addition, NP: combined N and P addition.

Fig. S2 Percentage of N remaining in leaf litter under different nutrient addition treatments in four species: (a-d) *Kobresia humilis*, (e-h) *Stipa aliena*, (i-l) *Tibetia himalaica*, and (m-p) *Gentiana straminea*. CK: control, N: nitrogen addition, P: phosphorus addition, NP: combined N and P addition.

Fig. S3 Percentage of P remaining in leaf litter under different nutrient addition treatments in four species: (a-d) *Kobresia humilis*, (e-h) *Stipa aliena*, (i-l) *Tibetia himalaica*, and (m-p) *Gentiana straminea.* CK: control, N: nitrogen addition, P: phosphorus addition, NP: combined N and P addition.

Changes in litter quality induced by nutrient addition alter litter decomposition in an alpine meadow on the Qinghai-Tibet Plateau

Wenyan Zhu, Jinzhou Wang, Zhenhua Zhang, Fei Ren, Litong Chen, JinSheng He

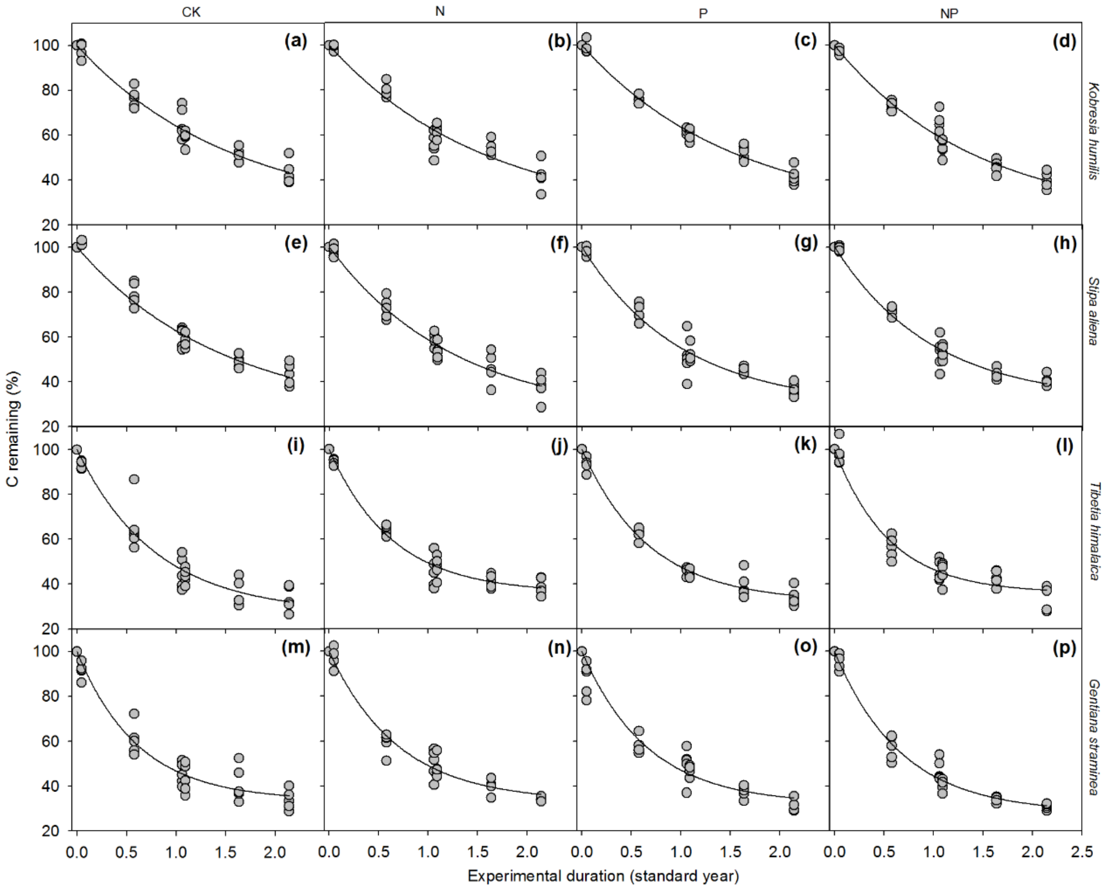

Fig. S1 Percentage of C remaining in leaf litter under different nutrient addition treatments in four species: (a-d) *Kobresia humilis*, (e-h) *Stipa aliena*, (i-l) *Tibetia himalaica*, and (m-p) *Gentiana straminea.* CK: control, N: nitrogen addition, P: phosphorus addition, NP: combined N and P addition.

Changes in litter quality induced by nutrient addition alter litter decomposition in an alpine meadow on the Qinghai-Tibet Plateau

Wenyan Zhu, Jinzhou Wang, Zhenhua Zhang, Fei Ren, Litong Chen, JinSheng He

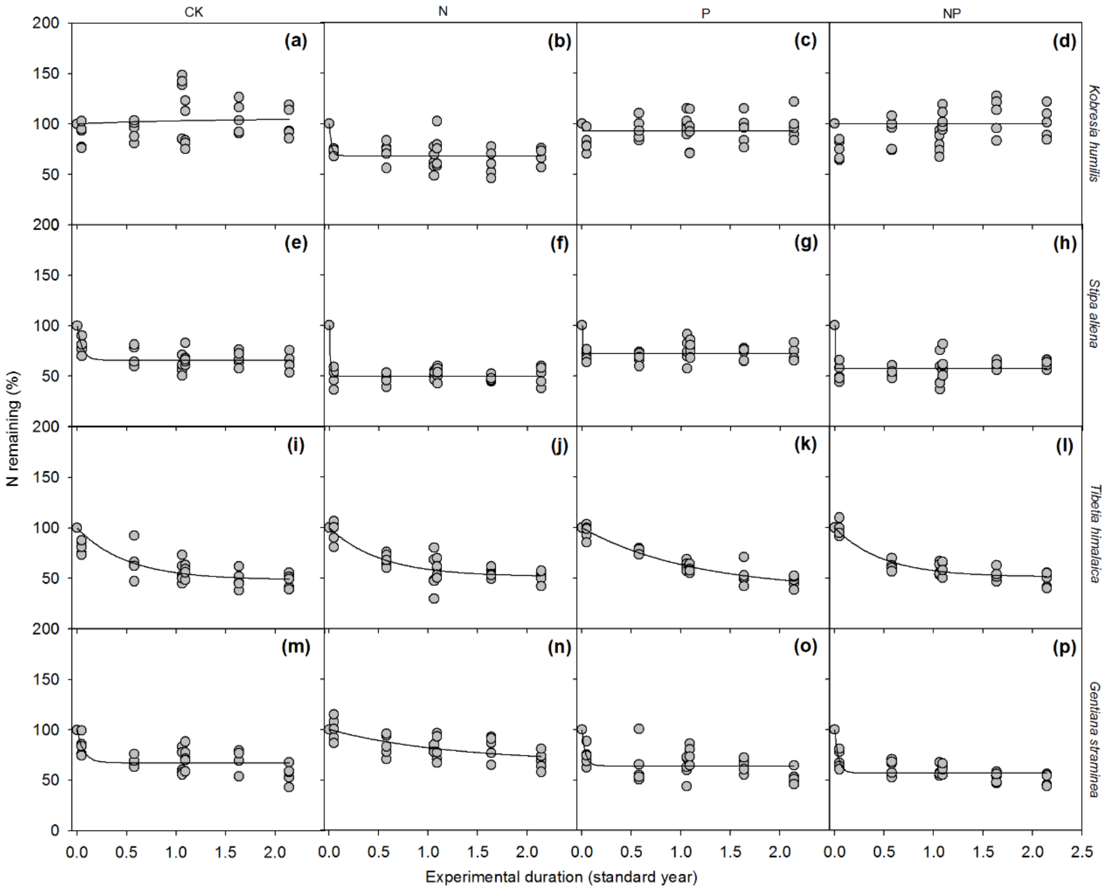

Fig. S2 Percentage of N remaining in leaf litter under different nutrient addition treatments in four species: (a-d) *Kobresia humilis*, (e-h) *Stipa aliena*, (i-l) *Tibetia himalaica*, and (m-p) *Gentiana straminea.* CK: control, N: nitrogen addition, P: phosphorus addition, NP: combined N and P addition.

Changes in litter quality induced by nutrient addition alter litter decomposition in an alpine meadow on the Qinghai-Tibet Plateau

Wenyan Zhu, Jinzhou Wang, Zhenhua Zhang, Fei Ren, Litong Chen, JinSheng He

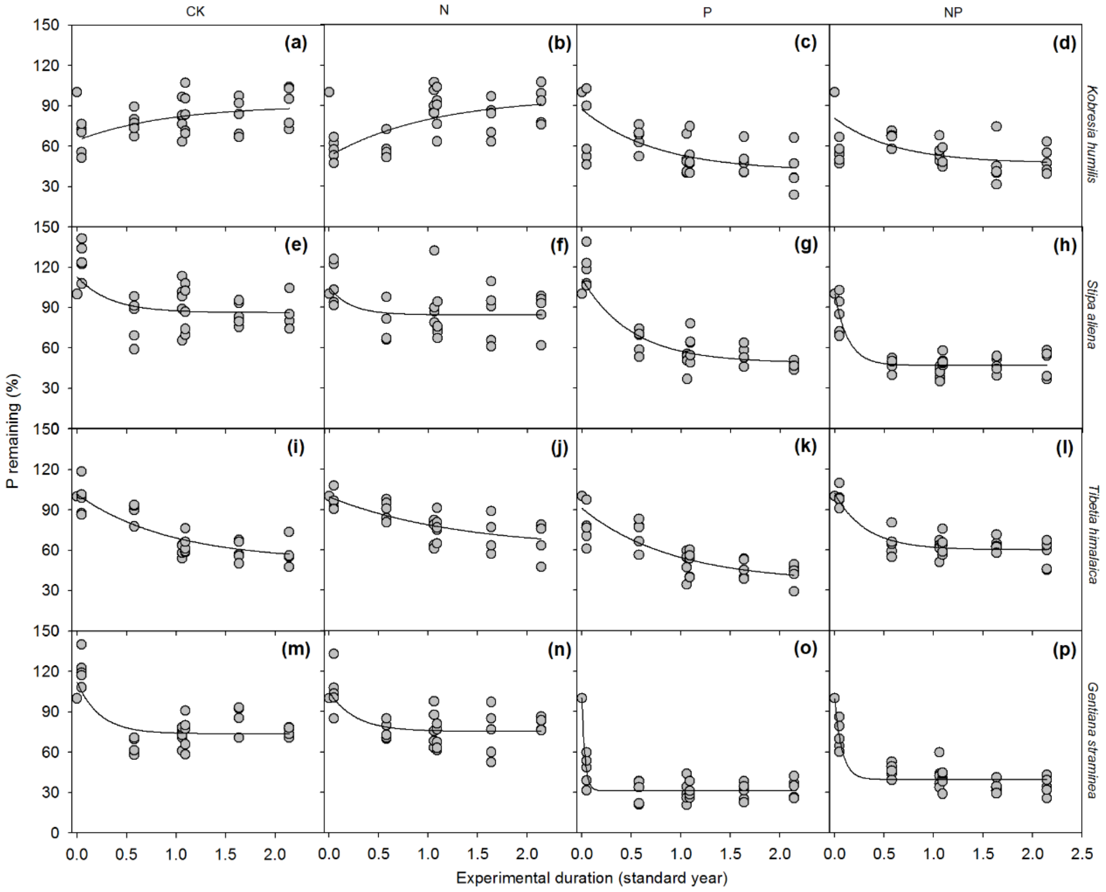

Fig. S3 Percentage of P remaining in leaf litter under different nutrient addition treatments in four species: (a-d) *Kobresia humilis*, (e-h) *Stipa aliena*, (i-l) *Tibetia himalaica*, and (m-p) *Gentiana straminea.* CK: control, N: nitrogen addition, P: phosphorus addition, NP: combined N and P addition.
